# The COVID-19 vaccination campaign in Bhutan: strategy and enablers

**DOI:** 10.1186/s40249-021-00929-x

**Published:** 2022-01-05

**Authors:** Tsheten Tsheten, Phurpa Tenzin, Archie C. A. Clements, Darren J. Gray, Lhawang Ugyel, Kinley Wangdi

**Affiliations:** 1 College of Health and Medicine, Research School of Population Health, Australian National University, 62 Mills Road, Acton, Canberra, ACT 2601 Australia; 2 Royal Centre for Disease Control, Ministry of Health, Thimphu, Bhutan; 3 Department of Public Health, Ministry of Health, Thimphu, Bhutan; 4 Telethon Kids Institute, Nedlands, Australia; 5 Curtin University, Perth, Australia; 6 School of Business, University of New South Wales, Canberra, ACT Australia

**Keywords:** Bhutan, Vaccine, COVID-19, Strategy, Campaign, Enablers, Report

## Abstract

Bhutan has reported a total of 2596 COVID-19 cases and three deaths as of September 15, 2021. With support from India, the United States, Denmark, the People’s Republic of China, Croatia and other countries, Bhutan was able to conduct two rounds of nationwide vaccination campaign. While many countries struggle to overcome vaccine refusal or hesitancy due to complacency, a lack of trust, inconvenience and fear, escalated in some countries by anti-vaccine groups, Bhutan managed to inoculate more than 95% of its eligible populations in two rounds of vaccination campaign. Enabling factors of this successful vaccination campaign were strong national leadership, a well-coordinated national preparedness plan, and high acceptability of vaccine due to effective mass communication and social engagement led by religious figures, volunteers and local leaders. In this short report, we described the national strategic plan and enabling factors that led to the success of this historical vaccination campaign.

## Background

Bhutan, a small landlocked country with a total area of 38,394 km^2^, is nestled in the Eastern Himalayas between India and China. The current population of the country is projected at 756,129 with a sex ratio of 110 males to 100 females. The majority (62%) of the population lives in rural areas. The median age is 26.5 years with an overall life expectancy of 70.3 years [1].

**Fig. 1:**
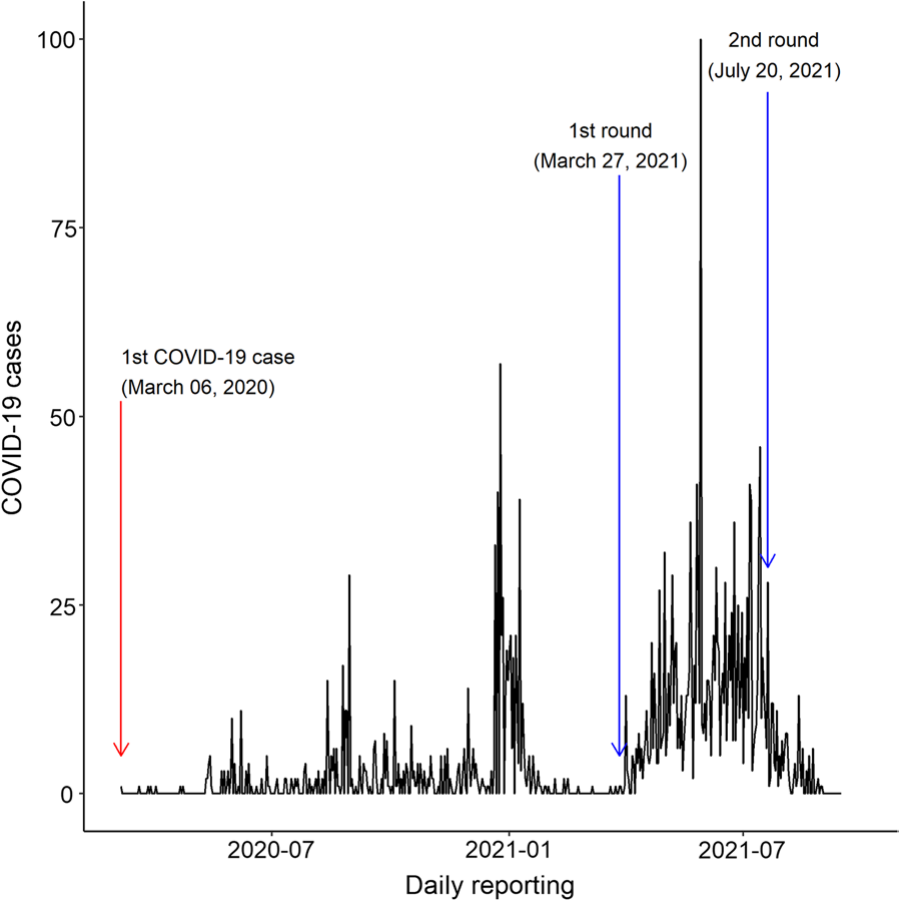
Daily reporting of COVID-19 cases from the beginning of the pandemic and the time period of nationwide vaccination rounds, Bhutan, 2021 ( Source: World Health Organization)

The coronavirus disease 2019 (COVID-19) in Bhutan was first confirmed on 5 March 2019, in a 76-year-old tourist [2]. Subsequently, there were sporadic cases and localized outbreaks in different parts of the country [3]. As of 15 September 2021, there were 2,596 confirmed cases and three deaths in the country (Fig. 1).

The vaccination program in Bhutan was introduced as a part of the global initiative to eradicate smallpox [4]. Whilst diphtheria, pertussis and tetanus (DPT), oral polio vaccine (OPV) and Bacillus Calmette-Guerin (BCG) vaccines were introduced in few districts in 1976, Bhutan launched its expanded programme on immunization (EPI) in 1979 to develop and expand immunization services to achieve Universal Childhood Immunization [4]. At present, Bhutan provides vaccines against tuberculosis, hepatitis B (hepB), poliomyelitis, diphtheria, tetanus, pertussis, haemophilus influenza type b (hib), measles, mumps and rubella. According to the 2020 EPI report, Bhutan has achieved more than 80% coverage for all vaccines in 20 districts without a single case of vaccine dropout for the pentavalent vaccine (DPT-hib-HepB) [5]. Recently, Bhutan expanded its national immunization schedule by introducing vaccines against human papilloma virus (2010), pneumonia (2019) and influenza (2020) [6].

Vaccination is one of the most cost-effective ways of preventing infectious diseases, currently saving 4–5 million deaths every year [7]. Notwithstanding the progress of vaccines, far too many people have insufficient access to vaccines particularly in developing countries due to inadequate resources [8]. To make the situation worse, vaccination coverage remains suboptimal due to the high level of vaccine hesitancy related to complacency, barriers to accessing vaccines, a lack of trust in government authorities, misinformation, and fear of adverse effects following immunization [9]. Here, we aimed to provide our perspectives on the drives that enabled high coverage of the COVID-19 vaccination campaign in Bhutan.

### Vaccination strategy

**Fig. 2:**
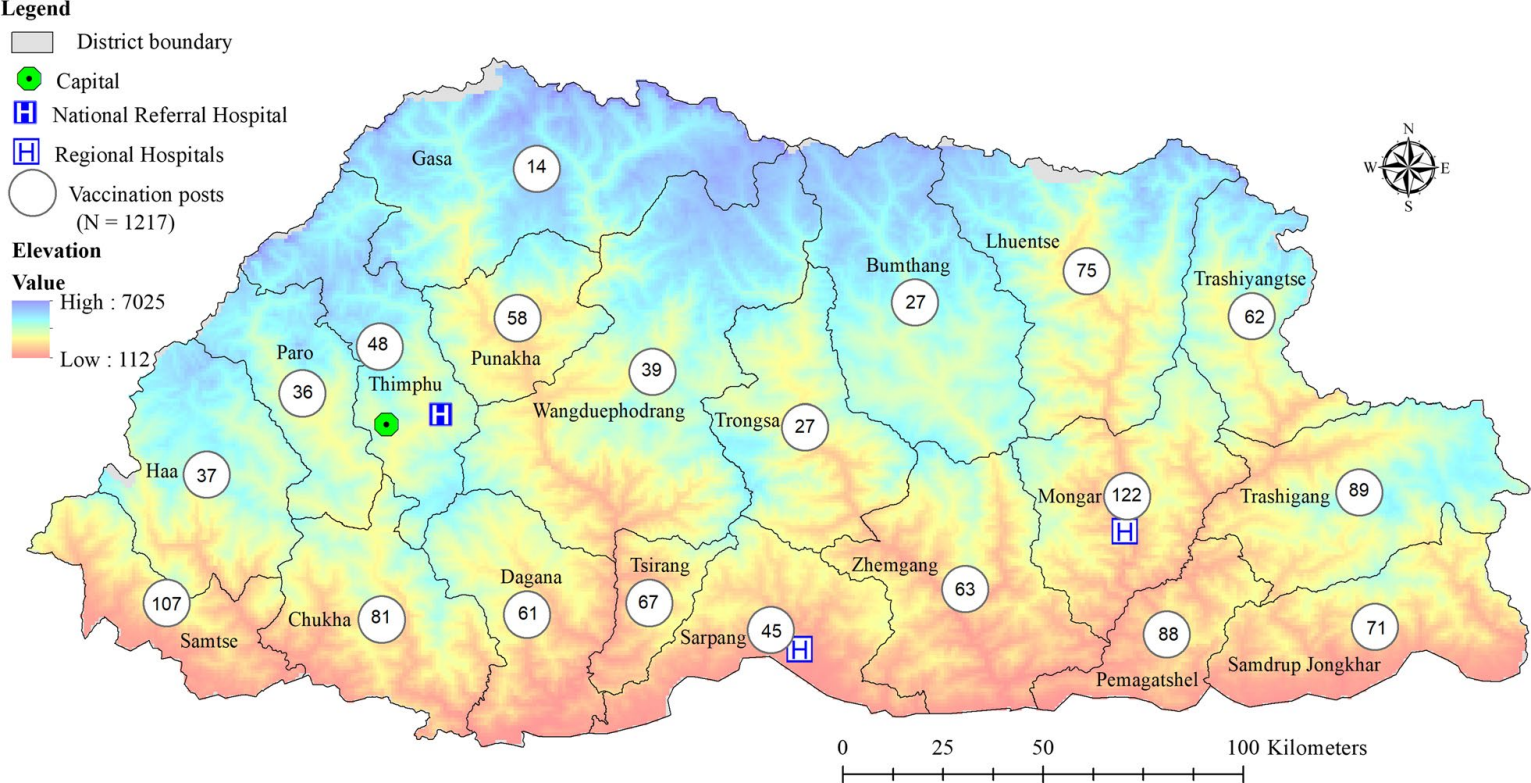
Distribution of vaccination posts/centres across 20 districts during the COVID-19 vaccination campaign in Bhutan, 2021. The figure in the circle indicates the number of vaccination posts set up in each district ( Source: Ministry of Health Facebook page)

Bhutan conducted its first nationwide COVID-19 vaccination round with the inoculation of the Covishield vaccine (Oxford-AstraZeneca) on 27 March 2021 coinciding with an auspicious day of the local astrological belief (Fig. 1). For the first round, Bhutan received a total of 550,000 doses of Covishield vaccine (Oxford-AstraZeneca) from India through the vaccine *Maitri* initiative as a goodwill gesture of friendship between the two countries. Of the total 496,044 eligible population aged ≥ 18 years, 478,829 were vaccinated across 1,217 vaccination centres in 3 weeks of the campaign, achieving a vaccination coverage of 96.5% [1, 10] (Fig. 2).

Due to a massive surge in cases and a shortage of vaccine supply in India, Bhutan sought support from other countries to provide vaccines, including the United States (500,000 doses Moderna and 5,850 doses of Pfizer-BioNTech vaccine), Denmark (250,000 doses of AstraZeneca vaccine), Croatia, Bulgaria and others (~ 100,000 doses of AstraZeneca vaccine), and the People’s Republic of China (50,000 doses of Sinopharm vaccine). Similar to the first COVID-19 vaccination round, the second round kicked off on the auspicious day of 20 July 2021 (Fig. 1). However, during this campaign, Bhutanese had the option to choose other brands such as Moderna, Pfizer-BioNTech, and Sinopharm vaccines. A cumulative total of 473,715 people were vaccinated within 2 weeks, covering 95.6% of the eligible adult population [11]. Of those vaccinated, 95% received heterologous vaccines and the remaining received homologous vaccines [12].

### Enablers of vaccine uptake

Enabling factors that led to high vaccination coverage in Bhutan are described below.

#### Strong leadership

The Prime Minister and the Health Minister guided and led the COVID-19 National and Regional Task Force committees responsible for planning and implementing all COVID-19 related initiatives. Perhaps, unique to Bhutan is the leadership role played by His Majesty the Fifth King, Jigme Khesar Namgyel Wangchuck. His Majesty worked together with the government in encouraging and inspiring the public to take COVID-19 vaccines. His Majesty has also visited the diverse geographical terrains in the mountainous northern borders and the hot and humid southern borders. During these royal visits, His Majesty visited and supervised vaccination posts to ensure vaccine toolkits and other necessary resources are put into place for the safe vaccination of the population.

#### Vaccination planning

Planning of the National vaccination campaign began soon after the start of the pandemic, at a time when vaccine trials were initiated in other countries. The Bhutan Vaccine System (BVS) (https://bvs.moh.gov.bt/) was developed and successfully implemented to digitally enumerate the eligible population for vaccination. In addition, BVS was used to select the number of vaccination posts, their locations and automatic generation of vaccine certificates for the vaccine recipients. BVS also provided an important platform to follow up with the registered individuals and encourage them for the vaccination program. This system is managed by the Ministry of Health (MoH) and is user-friendly, leading to a high proportion of people being registered in it.

For the elderly and those individuals with mobility issues, home-based vaccinations were arranged. To overcome the physical barriers of rugged, mountainous terrain and to maintain a proper cold chain of the vaccine during the transportation process, vaccination services were facilitated by the Royal Bhutan Airlines and the Bhutan Helicopter Services Limited. This meant that vaccines were available in all the vaccination posts.

#### Vaccine communication

A massive public education programme was undertaken using appropriate vaccine communication strategies including pamphlets, advertisements on the national television channel (BBSTV) and radio, press briefs, and notices on the Facebook page of the MoH and the Prime Minister’s Office (PMO). The Prime Minister, Foreign Minister, and Health Minister regularly provided updates to alleviate any fear of vaccination. Further, the benefits of COVID-19 vaccination were discussed on BBSTV by vaccine experts and epidemiologists.

#### Health and religion

The Central Monk Body of Bhutan (*Zhung Dratshang*) and other monastic organizations led by spiritual masters (*Rinpoches*) played a pivotal role in building trust in COVID-19 control through vaccination and other means. The specific times of the vaccination rounds were fixed according to the advice of the *Zhung Dratshang* based on astrological beliefs. Through religious discourse and teachings, these organizations were able to inspire people with otherwise anti-vaccine sentiments, along with the population at large, to accept the vaccine.

#### Social engagement

A large volunteer workforce, known locally as *Desuups* (www.desuung.org.bt), came forward to facilitate the organization of vaccination rounds and other activities to control COVID-19 [3]. Founded by the Fifth King, *Desuup* trainees undergo a value-based personal development program to encourage volunteerism for community services and play an active role in building the nation. During the vaccination campaign, *Desuups* were deployed in every vaccination post and supported conducting online registration and verification of vaccine recipients, and ensuring compliance with the COVID-19 safety protocol. The armed forces, foresters and customs officials were also deployed to support the vaccination campaign as well as during the COVID-19 pandemic to maintain law and order in strategic locations such as crowded places and along the border to prevent illegal immigrants. It is interesting to note that many people, after receiving the vaccine, described their positive experiences on their social media platforms and the MoH web page, encouraging people to get vaccinated.

There are a few limitations worth noting in this study. Firstly, opinions expressed in this study could have been influenced by the researcher’s perception and understanding of the vaccination campaign. Secondly, inferences were based on the vaccination data from open sources such as the Facebook page of the MOH and the PMO. Authors believe these are credible sources with reliable information.

## Conclusions

Adequate vaccination is the most important long-term solutions in the fight against COVID-19. Bhutan’s high coverage of COVID-19 vaccine is attributed to strong leadership, a well-coordinated national preparedness plan, regular communication strategies and successful social mobilization. Despite high vaccination coverage, the COVID-19 containment measures such as social distancing and handwashing have to be continued to limit the spread of COVID-19, particularly in the context of new emerging coronavirus variants and low vaccine coverage in neighbouring countries. Additional rounds, including booster doses, might be necessary for the continued protection of the population.

## Data Availability

The dataset used and in the current study were available on the WHO website (https://covid19.who.int/table).

## Abbreviations

BBS-TV: Bhutan Broadcasting Service Television
BVS: Bhutan Vaccine System
COVID-19: Coronavirus disease 2019
MoH: Ministry of Health
PMO: Prime Minister Office
UNDP: United Nations Development Programme
VHW: Village Health Worker
WHO: World Health Organization
